# Temporal Viral Genome-Protein Interactions Define Distinct Stages of Productive Herpesviral Infection

**DOI:** 10.1128/mBio.01182-18

**Published:** 2018-07-17

**Authors:** Jill A. Dembowski, Neal A. DeLuca

**Affiliations:** a Department of Microbiology and Molecular Genetics, University of Pittsburgh School of Medicine, Pittsburgh, Pennsylvania, USA; University of Michigan—Ann Arbor

**Keywords:** DNA damage, DNA repair, ICP4, affinity purification, herpes simplex virus, iPOND, mediator, transcription

## Abstract

Herpesviruses utilize multiple mechanisms to redirect host proteins for use in viral processes and to avoid recognition and repression by the host. To investigate dynamic interactions between herpes simplex virus type 1 (HSV-1) DNA and viral and host proteins throughout infection, we developed an approach to identify proteins that associate with the infecting viral genome from nuclear entry through packaging. To accomplish this, virus stocks were prepared in the presence of ethynyl-modified nucleotides to enable covalent tagging of viral genomes after infection for analysis of viral genome-protein interactions by imaging or affinity purification. Affinity purification was combined with stable isotope labeling of amino acids in cell culture (SILAC) mass spectrometry to enable the distinction between proteins that were brought into the cell by the virus or expressed within the infected cell before or during infection. We found that input viral DNA progressed within 6 h through four temporal stages where the genomes sequentially (i) interacted with intrinsic antiviral and DNA damage response proteins, (ii) underwent a robust transcriptional switch mediated largely by ICP4, (iii) engaged in replication, repair, and continued transcription, and then (iv) transitioned to a more transcriptionally inert state engaging *de novo*-synthesized viral structural components while maintaining interactions with replication proteins. Using a combination of genetic, imaging, and proteomic approaches, we provide a new and temporally compressed view of the HSV-1 life cycle based on input genome-proteome dynamics.

## IMPORTANCE

Herpesviruses are highly prevalent and ubiquitous human pathogens. Studies of herpesviruses and other viruses have previously been limited by the ability to directly study events that occur on the viral DNA throughout infection. We present a new powerful approach, which allows for the temporal investigation of viral genome-protein interactions at all phases of infection. This work has integrated many results from previous studies with the discovery of novel factors potentially involved in viral infection that may represent new antiviral targets. In addition, the study provides a new view of the HSV-1 life cycle based on genome-proteome dynamics.

## INTRODUCTION

Herpesviruses are a family of highly prevalent eukaryotic viruses that share strong evolutionary relationships with their hosts (1). They have therefore developed sophisticated mechanisms to invade host cells, alter cellular activities, and redirect host factors for use in viral processes. Knowledge of how herpesviruses manipulate the host to evade intrinsic responses to infection or to utilize host cell resources to drive productive infection or the establishment of latency is crucial for understanding their life cycles.

Herpes simplex virus type 1 (HSV-1) is a ubiquitous human pathogen that infects the majority of the human population. Initial productive infection occurs in epithelial cells where the viral genome is prolifically transcribed and replicated, resulting in many new virus progeny. The virus can also gain access to sensory neurons, where it can undergo productive infection or establish reversible latency (2). During latency, most of the viral genome is transcriptionally repressed, and no progeny are produced. Thus, processes that occur on the viral genome largely determine the outcome of infection.

Nuclear stages of productive infection involve coordinated events occurring on the viral genome, which begin with the transfer of viral DNA into the host nucleus through the nuclear pore shortly after infection (3). Once in the nucleus, the viral genome is subject to the opposing actions of the intrinsic antiviral response mediated at promyelocytic leukemia (PML) nuclear bodies (NBs), and the counteracting functions of viral proteins, particularly ICP0 (4, 5). A coordinated and sequential cascade of expression of three temporal classes of viral genes ensues (6, 7). Transcription of immediate early (IE) viral genes is activated by the viral tegument protein VP16 (8, 9), transcription of early and late viral genes is activated by the IE gene product ICP4 (10–12), and transcription of late viral genes is coupled to viral DNA replication by an unknown mechanism. IE gene products include regulatory proteins, early gene products include the viral replication machinery, and late gene products mostly comprise the structural components of the virus (6, 7). Replicated DNA is packaged into preassembled capsids, which subsequently exit the nucleus. How these events are staged with respect to the actions of viral and cellular protein complexes acting on the viral genome is unclear. This is mostly due to issues of sensitivity in the measurements of processes occurring during single step growth, as well as the fact that time of occurrence of crucial events can be obscured by events that are more quantitatively robust.

To examine events that occur on viral genomes, we previously developed an approach based on isolation of proteins on nascent DNA (iPOND) (13) to selectively label replicating viral DNA within infected cells with ethynyl-modified nucleotides (5-ethynyl-2′-deoxycytidine [EdC] or 5-ethynyl-2′-deoxyuridine [EdU]) to enable the covalent conjugation to biotin-azide or Alexa Fluor-azide (14). Biotinylated DNA is purified on streptavidin-coated beads followed by the identification of associated proteins by mass spectrometry (MS). Furthermore, Alexa Fluor-modified genomes can be imaged in cells relative to specific host or viral proteins. These approaches were used to establish spatiotemporal relationships between specific viral and cellular proteins and the replicating HSV-1 genome and reveal the potential involvement of host factors in processes that occur on nascent viral DNA during relatively late stages of productive infection (14, 15). These and other studies also demonstrate that it is possible to track infecting viral genomes that have been prelabeled with ethynyl-modified nucleotides by imaging approaches (14, 16–18).

Herein we used viral genome purification and imaging approaches to investigate dynamic changes that occur on the original infecting viral genome during distinct stages of infection. HSV-1 structural and tegument proteins from the infecting virus are associated with the infecting viral genome early during infection. As infection proceeds, the same structural proteins are synthesized in the infected cell and then again become associated with viral genomes. Therefore, we utilized stable isotope labeling of amino acids in cell culture (SILAC) to differentiate between proteins that originate in the infecting virion and proteins that were synthesized within the infected cell. Additionally, we performed this analysis on both wild-type virus and a virus that does not synthesize ICP4 to investigate changes that mediate a robust transcriptional switch early during infection. Using a combination of genetic, imaging, and proteomic approaches, we have tracked the fate of input viral genomes within the nuclei of host cells throughout the course of infection and defined several crucial steps that occur early in the productive HSV-1 life cycle.

## RESULTS

### Input viral genomes can be tracked from nuclear entry through packaging.

To investigate the protein landscape associated with input viral DNA, wild-type HSV-1 (KOS) stocks were prepared in the presence of EdC to label viral genomes, which enables subsequent imaging or purification of input viral DNA after infection. EdC labeling resulted in an approximately threefold increase in the genome/PFU ratio (see Table S1 in the supplemental material), suggesting that incorporation of EdC into viral DNA results in a modest decrease in infectivity. This is likely a result of incomplete or incorrect assembly of infectious particles.

10.1128/mBio.01182-18.6 TABLE S1  Effects of EdC labeling on viral genome/PFU ratio. Virus stocks were prepared in the presence or absence of EdC (final concentration of 10 µM EdC for KOS and final concentration of 5 µM EdC for n12]), and genome number and PFU were determined by real-time PCR and plaque assay, respectively. n12 virus stocks were prepared, and the titers of the virus in the ICP4-complementing cell line E5 were determined. Values indicate the number of genomes or PFU per µl of virus stock. Download TABLE S1, DOC file, 0.01 MB. Copyright © 2018 Dembowski and DeLuca. 2018 Dembowski and DeLuca This content is distributed under the terms of the Creative Commons Attribution 4.0 International license.

**FIG 1:**
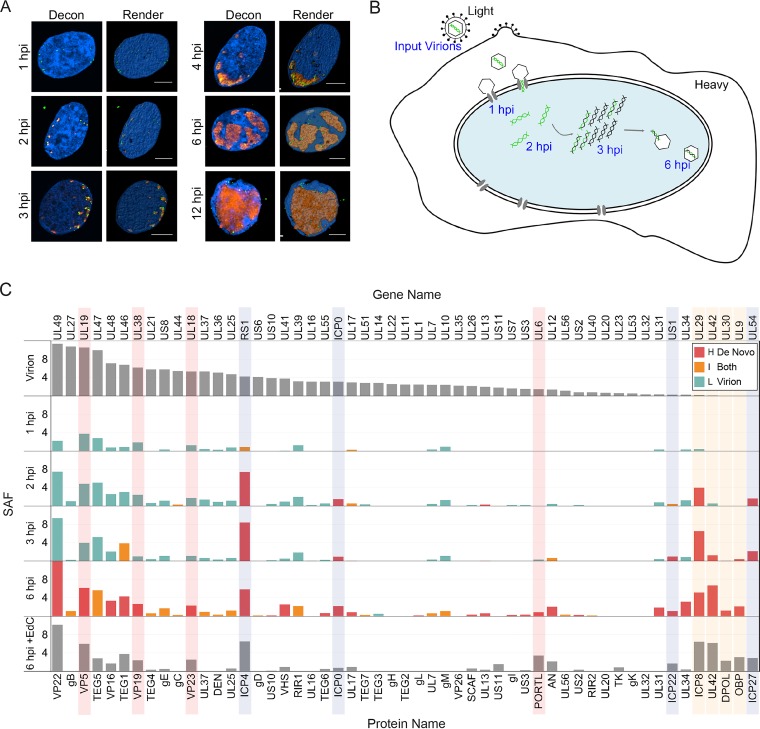
The fates of input viral genomes can be tracked within infected-cell nuclei. (A) Visualization of input viral DNA. Cells were fixed at the indicated times after infection with KOS-EdC (1 to 12 hpi) at an MOI of 10 PFU/cell. Viral genomes (green) and ICP4 (red) were imaged relative to host nuclei (blue). Optical sections were deconvolved (Decon) to generate high-resolution images and rendered (Render) to illustrate the positions of input viral DNA. Bars, 5 µm. (B) Model depicting the fate of input viral genomes during distinct stages of infection. Input viral DNA is shown in green, and replicated viral DNA is shown in black. For MS experiments, input virions and the infected cell contain proteins labeled with light or heavy amino acids, respectively. (C) Abundance of viral-genome-associated viral proteins throughout infection. Viral-genome-associated viral proteins were detected by MS after a 1-, 2-, 3-, or 6-h infection with KOS-EdC (MOI of 10 PFU/cell). Spectral abundance factors (SAFs) were plotted relative to individual proteins associated with mature virions (Virion) and viral replication compartments that were labeled with EdC from 4 to 6 hpi (6 hpi + EdC) (15). Proteins were distinguished as either heavy (H), light (L), or intermediate (I) by SILAC analysis. In cases where SILAC analysis was not carried out, bar graphs are shown in gray. IE viral proteins are shown on a light gray background, viral replication proteins on tan background, and capsid proteins on pink background. Data represent results from one of two biological replicates. See also Tables S1 and S2 and Fig. S1 and S2 in the supplemental material.

To demonstrate the sensitivity and specificity of viral genome labeling, Vero cells were infected with EdC-labeled KOS (KOS-EdC) and fixed at various times after infection. Fixed cells were subject to click chemistry and immunofluorescence to visualize the relative location of input viral DNA and ICP4 within the host nuclei (Fig. 1A). At 1 h postinfection (hpi), viral genomes were observed at the perimeter of the nuclear membrane as they entered into the nucleus through the nuclear pore. By 2 hpi, ICP4 was expressed and colocalized with most, if not all, viral genome foci. From 3 to 12 hpi, after the onset of viral DNA replication, ICP4 foci representing replication compartments grew in size, while input genomes contained within these foci could still be distinguished as discrete puncta. At later times, input viral DNA appeared to coalesce and migrate to the perimeter of replication compartments. One explanation for this is that fragments of input genomes that underwent replication and recombination were condensed for repackaging into nascent capsids. Alternatively, these foci may contain genomes that were repressed at the onset of infection. Together these data demonstrate the specificity of the click chemistry approach for tracing the fate of the input viral genome throughout the course of infection (Fig. 1B).

To investigate the ordered protein interactions that occur on input viral DNA, human MRC-5 fibroblast cell nuclei were harvested at specific times after infection with KOS-EdC, and viral DNA was covalently attached to biotin and purified using streptavidin-coated beads followed by mass spectrometry (MS) to identify associated proteins (Fig. 1C; Table S2). To compare the relative abundance of identified proteins, spectral abundance factors (SAFs) (spectral counts/molecular weight) were calculated for each protein and plotted in order of relative abundance in mature virions (Fig. 1C, Virion). For comparison, proteins found to associate with viral replication compartments were also graphed (Fig. 1C, 6 hpi + EdC) (19). Stable isotope labeling of amino acids in cell culture (SILAC) was performed to distinguish between factors that originated in the infecting virion (light amino acids) and factors that were expressed within the infected cells either before or during infection (heavy amino acids). The relative intensities of amino acids were compared (Fig. 1C), and input genome-associated proteins were distinguished as heavy (H), light (L), or intermediate (I) and to have therefore originated in the infected cell, virion, or both. SILAC MS analysis of viral proteins was highly reproducible (see Fig. S1A in the supplemental material).

10.1128/mBio.01182-18.1 FIG S1  Viral protein identification and SILAC analysis are highly reproducible. (A) Viral-genome-associated viral proteins were detected by MS after a 1-, 2-, 3-, or 6-h infection with KOS-EdC or n12-EdC, and SAFs of individual proteins were plotted. Proteins were distinguished as either heavy (H) (red), light (L) (teal), or intermediate (I) (orange) by SILAC analysis. Biological replicates reveal the reproducibility of both protein identification and SILAC MS. (B) Nascent capsid proteins associate with input genomes in roughly the same relative abundance as they constitute intact capsids at 6 hpi. The abundance of capsid proteins within B capsids was determined previously (21, 22), and the graphed values correspond to the number of copies of the protein multiplied by the molecular weight. The intensity of heavy peptides was determined by SILAC analysis of capsid proteins found to associate with input viral genomes at 6 hpi. VP26 was not detected in these studies. Download FIG S1, TIF file, 2.6 MB. Copyright © 2018 Dembowski and DeLuca. 2018 Dembowski and DeLuca This content is distributed under the terms of the Creative Commons Attribution 4.0 International license.

10.1128/mBio.01182-18.7 TABLE S2  MS and SILAC data. Download TABLE S2, XLSX file, 0.2 MB. Copyright © 2018 Dembowski and DeLuca. 2018 Dembowski and DeLuca This content is distributed under the terms of the Creative Commons Attribution 4.0 International license.

At 1 hpi, viral genomes associated with light capsid and tegument proteins that were brought into the cell with the infecting virion. Immediate early viral gene products (ICP0, ICP4, ICP22, and ICP27) are expressed by 1 hpi (20) and were found to associate with infecting viral genomes by 2 hpi (Fig. 1C, shown on light gray background). ICP8 was the first viral replication factor to associate by 2 hpi, and additional replication factors were detected by 3 hpi (UL42, UL30, and UL9, shown on tan background), the time at which viral genomes begin to replicate (15). Replication factors are not abundant in the virion and were therefore expressed *de novo* subsequent to infection and generally contained heavy amino acids. At later times (6 hpi), viral genomes were found to associate with newly expressed viral structural proteins, including capsid proteins VP5, VP19, VP23, and UL6 (Fig. 1C, shown on pink background). A clear transition from light to heavy capsid proteins was observed by 6 hpi, and nascent capsid proteins associated with these genomes in roughly the same relative abundance as they constitute intact capsids (Fig. S1B) (21, 22). These data suggest that some population of the input viral DNA was repackaged by this time.

Costaining of input genomes, ICP4, and the major viral capsid protein (VP5) demonstrates that labeled input genomes are released from capsids docked at the nuclear membrane at early stages of infection (1 hpi) (Fig. S2). These genomes associate with ICP4 by 2 hpi (Fig. S2). Nascent VP5, a late gene product, accumulates in the nucleus by 4 hpi (Fig. S2). Input genomes colocalize with VP5 associated with replication compartments by 6 hpi (Fig. S2). Taken together, temporal viral genome-viral protein interactions observed in these studies are consistent with known events in the virus life cycle and demonstrate the sensitivity, specificity, and reproducibility of the input viral genome purification approach.

10.1128/mBio.01182-18.2 FIG S2  Localization of the HSV-1 major capsid protein relative to input viral DNA and ICP4 throughout infection. Cells were fixed at the indicated times after infection with KOS-EdC (1 to 6 hpi) or mock infection. Viral genomes (green), ICP4 (blue), and the major capsid protein VP5 (red) were imaged relative to host nuclei (dashed lines). Arrows indicate the positions of input viral DNA. The inset or box in the corner of each image includes a 3.5× zoomed-in image of the region indicated by the arrowhead. Download FIG S2, TIF file, 1.4 MB. Copyright © 2018 Dembowski and DeLuca. 2018 Dembowski and DeLuca This content is distributed under the terms of the Creative Commons Attribution 4.0 International license.

### Host proteins associate with input genomes upon nuclear entry.

Host proteins identified to associate with affinity-purified input genomes are listed in Table S2. To further demonstrate the reproducibility of this assay to determine the relative abundance of factors associated with viral DNA, the SAF values of individual proteins from duplicate experiments were plotted to determine the Pearson correlation coefficient of replicate experiments (Fig. S3). In all cases, the correlation coefficient was at least 0.92, demonstrating a linear relationship between data points and a general consistent trend in the relative yield of individual factors using this approach. Although many experimental variables govern whether a protein will be captured and identified using this technique, results are consistent between replicate experiments, and there is high confidence in the presence of identified factors.

10.1128/mBio.01182-18.3 FIG S3  MS analysis is reproducible. Comparison of the SAFs of individual proteins found to associate with viral genomes at 1, 2, 3, or 6 hpi with KOS-EdC or n12-EdC. Each point represents an individual protein with the SAF from the first biological replicate plotted on the *x* axis and the SAF from the second biological replicate plotted on the *y* axis. The linear regression line is shown for reference, and r represents the calculated Pearson correlation coefficient, which indicates the similarity between replicate experiments. Sample KOS + EdC 6 hpi includes previously published data in which replicating viral DNA was labeled with EdC from 4 to 6 hpi to enable the subsequent purification of nascent viral DNA (19). Download FIG S3, TIF file, 1.9 MB. Copyright © 2018 Dembowski and DeLuca. 2018 Dembowski and DeLuca This content is distributed under the terms of the Creative Commons Attribution 4.0 International license.

No host factors were reproducibly found to contain peptides labeled with light amino acids. Therefore, we conclude that cellular proteins associated with viral genomes at early stages of infection do not originate from the infecting virus particle. Potential interactions among viral-genome-associated host factors identified by MS were illustrated using the STRING protein-protein interaction network database (23). One hour after infection, host proteins identified to associate with viral DNA include the catalytic subunit of host polymerase (Pol) II (POLR2A), factors that play roles in transcription regulation and RNA processing (INTS1, USP39, SRRT, DDX23, and THOC7), core components of PML NBs (PML, SP100, and SUMO2), factors involved in the regulation of chromatin structure (HP1BP3, HIST1H1A, HIST1H1E, CHD4, CSNK2A1, SUPT16H, SMARCC2, SMARCA4, and TRRAP), and factors that are recruited to damaged DNA (PARP1, PARP14, RPA1, and ligase 3 [LIG3]). Identified host factors illustrate the processes that occur on HSV-1 genomes shortly after entry into the nucleus: (i) transcription of IE viral genes, (ii) association with promyelocytic leukemia (PML) nuclear bodies (NBs) and components of cellular chromatin, and (iii) recognition by the host cell as DNA damage.

**FIG 2:**
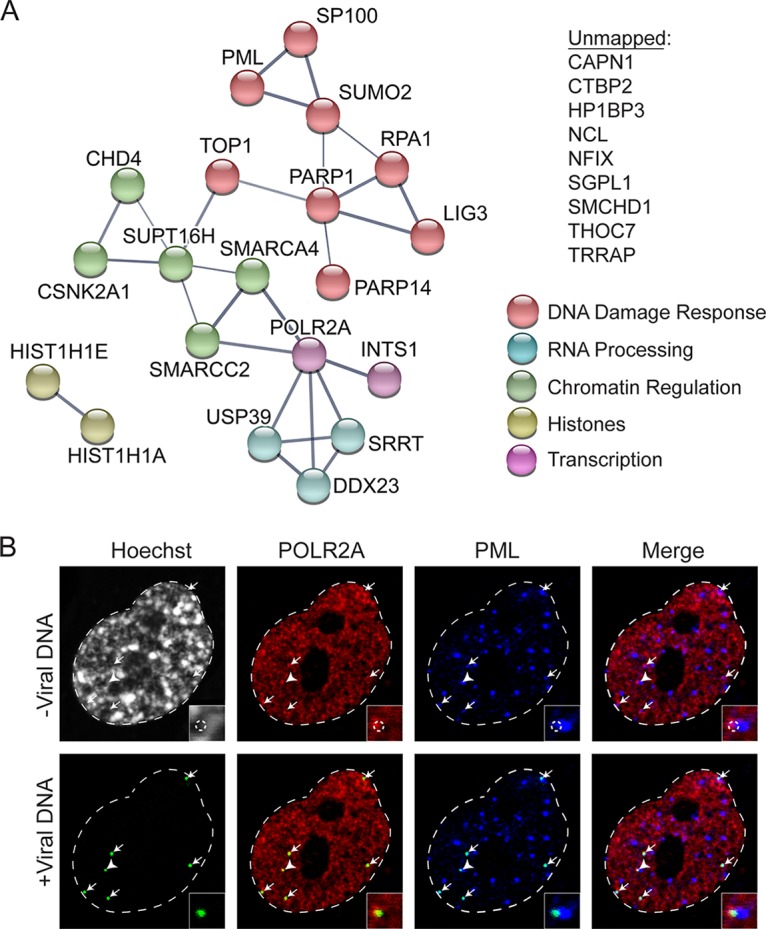
Host DNA damage response factors and Pol II associate with input viral genomes by 1 hpi. (A) Graphic illustration of predicted physical and functional interactions between human proteins that associate with HSV-1 genomes at 1 hpi with KOS-EdC (MOI of 10 PFU/cell). The biological processes in which identified proteins are likely involved are indicated by different colors, and the unmapped list includes proteins that were not mapped using STRING. (B) Input viral genome foci simultaneously associate with Pol II and PML. MRC-5 cells were infected with KOS-EdC at an MOI of 10 PFU/cell, fixed at 1 hpi, and subjected to click chemistry to label input viral DNA (green), Hoechst staining to label nuclei, and indirect immunofluorescence to label Pol II (POLR2A [red]) and PML (blue). All panels represent different views of the same nucleus, which is outlined in white. White arrows indicate the locations of input viral genomes, and the insets or corner boxes show a 3× zoomed-in image of the area indicated by the white arrowhead. Viral DNA was omitted from the top panel (-Viral DNA), and the location of input viral genome foci is outlined by a dashed circle in the zoomed-in image. Viral DNA foci were included in the bottom panel (+Viral DNA). See also Fig. S3 and Table S2.

To determine whether multiple processes occur on each genome or whether these results reflect the existence of mixed populations of viral DNA engaged in different processes at 1 hpi, we carried out costaining for a protein involved in viral repression (PML) and the core subunit of Pol II (POLR2A) (Fig. 2B). We demonstrate that individual viral genome foci are associated with both PML and Pol II but that PML and Pol II do not colocalize with each other. This is consistent with the observation that viral genomes are juxtaposed to PML NBs at this time (24). We cannot distinguish between whether individual foci contain more than one genome. However, because these foci appear to originate from single capsid foci (Fig. S2), we hypothesize that the viral genome foci represent individual genomes at early times postinfection (1 to 2 hpi). Taken together, viral genomes are recognized by the cell as DNA damage early during infection and are associated with PML NBs. At the same time, portions of the viral genome are capable of interacting with Pol II, likely to enable transcription of IE viral genes.

### Robust transcription factor recruitment to viral genomes occurs coincident with the binding of ICP4.

**FIG 3:**
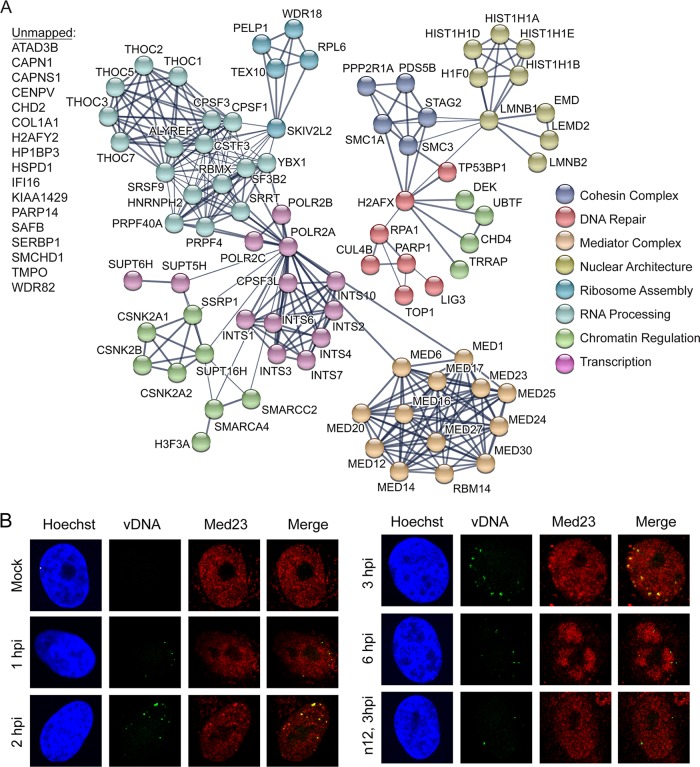
Robust recruitment of host transcription factors to input viral DNA. (A) Illustration of predicted physical and functional interactions between human proteins that associate with HSV-1 genomes at 2 hpi with KOS-EdC (MOI of 10 PFU/cell). (B) Input viral genome foci associate with Med23 starting around 2 hpi. MRC-5 cells were infected with KOS-EdC or n12-EdC at an MOI of 10 PFU/cell, fixed at the indicated times, and subject to click chemistry to label input viral DNA (vDNA) (green), Hoechst staining to label nuclei (blue), and indirect immunofluorescence to label Med23 (red). See also Fig. S3 and Table S2.

After 2 h, PML NBs are dispersed through the actions of ICP0, and ICP4 associates with the viral genome to activate transcription of early viral genes. In this study, nascent ICP4 was found to associate with input viral genomes (Fig. 1C), and PML components (PML, SP100, and SUMO2) were no longer detected by 2 hpi (Fig. 3A). At this time, several host factors involved in host cell transcription were found to associate with viral DNA. These factors include components of the Mediator (MED1, -6, -12, -14, -16, -17, -20, -23, -24, -25, and -27) and Integrator (INTS1, -2, -3, -4, -6, -7, and -10 and CPSF3L) complexes, as well as factors that regulate transcription elongation (SSRP1, SPT16H, SUPT5H, and SUPT6H) and RNA processing. With the exception of POLR2A, INTS1, and THOC7, associated transcription and RNA processing factors were not detected before 2 h and were therefore potentially recruited through the actions of ICP4, another IE viral gene product, or as a result of alterations in viral genome architecture. It is likely that small amounts of some of these complexes are recruited to the genome earlier through the action of VP16 but are present below the limits of detection. We previously demonstrated that ICP4 interacts with the Mediator complex and was required for the recruitment of Mediator components to viral promoters (25). Furthermore, the IE gene product ICP22 is required for the recruitment of SSRP1 to viral DNA (26). To verify the timing of recruitment of Mediator to viral DNA, we costained infected cells for infecting viral genomes and the Mediator component Med23 (Fig. 3B). Med23 did not colocalize with viral genomes by 1 hpi but did colocalize by 2 hpi, validating the MS results.

Furthermore, by 2 hpi, TP53BP1 and IFI16 were also found to associate with viral genomes, as well as components of the nuclear lamina (LEMD2, EMD, LMNB1, and LMNB2) and the cohesin complex (SMC1A, SMC3, STAG2, and PDS5B). Interestingly, the nuclear lamina may play a role in the reduction of heterochromatin on viral genes (27). Taken together, there is an obvious switch in viral genome architecture that occurs between 1 and 2 hpi that likely mediates the onset of early viral gene expression and sets the stage for viral genome replication.

### ICP4 facilitates transcription factor recruitment.

To investigate the role ICP4 plays in the recruitment of host transcription factors to viral DNA, viral stocks of the ICP4 mutant n12 (28) were prepared by propagating the virus in the presence of EdC. EdC labeling of n12 in the ICP4-complementing cell line E5 resulted in a two-fold increase in the genome/PFU ratio (Table S1). Therefore, as observed for wild-type KOS, viral genome labeling resulted in a modest decrease in n12 infectivity. To verify that EdC labeling was specific, EdC-labeled n12 (n12-EdC)-infected cells were subjected to immunofluorescence (Fig. S4). Viral genomes were observed at the perimeter of the nucleus at 3 hpi (n12, Vero, 3 hpi) and did not progress to form replication compartments unless ICP4 was supplied in *trans* (n12, E5, 6 hpi) (Fig. S4). Therefore, the analysis of n12-EdC infection should enable the investigation of changes that occur on the viral genome as a consequence of ICP4 association.

10.1128/mBio.01182-18.4 FIG S4  EdC-labeled n12 viral genomes can be visualized within infected-cell nuclei and form replication compartments when ICP4 is supplied in *trans*. Vero or E5 cells were infected with KOS-EdC or n12-EdC and were fixed for imaging at 3 or 6 hpi. Viral genomes (green) and ICP4 (red) were imaged relative to host nuclei (blue). Download FIG S4, TIF file, 2.4 MB. Copyright © 2018 Dembowski and DeLuca. 2018 Dembowski and DeLuca This content is distributed under the terms of the Creative Commons Attribution 4.0 International license.

**FIG 4:**
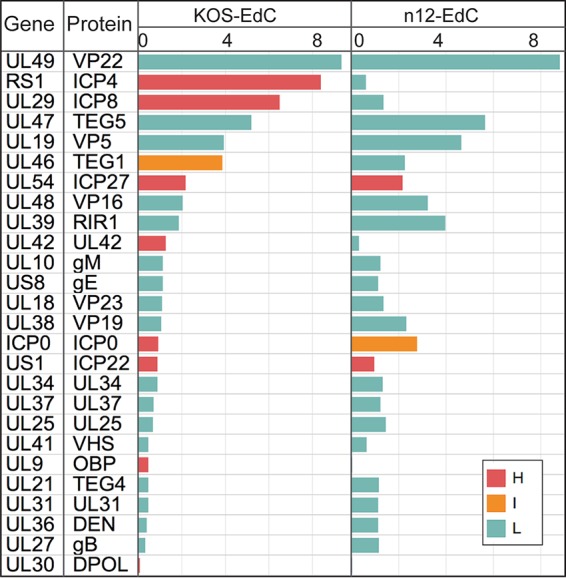
ICP4 is required for the expression and subsequent association of early, but not IE, viral gene products with input viral genomes. Viral-genome-associated viral proteins were detected by MS at 3 hpi with KOS-EdC or n12-EdC (MOI of 10 PFU/cell). Proteins were distinguished as either heavy (H), light (L), or intermediate (I) by SILAC analysis. See also Tables S1 and S2 and Fig. S1, S4, and S5.

MRC-5 cells were infected with n12-EdC, and proteins that associated with the genome at 3 hpi were determined as described above. Identified viral proteins were graphed relative to viral proteins found to associate with KOS-EdC viral genomes at this time (Fig. 4). A small amount of ICP4 was purified with n12 viral genomes. We conclude that this population of ICP4 was carried into the cell as part of the viral tegument because it was enriched in light amino acids. MS of purified virions also revealed that ICP4 is a component of mature n12 virions, which contain the same protein composition as KOS virions (Fig. S5). n12-infected cells do not efficiently express early viral genes, including ICP8, UL42, UL9, and UL30, and as a consequence, these proteins were not abundantly associated with n12 viral genomes which do not undergo viral DNA replication in noncomplementing cells (28).

10.1128/mBio.01182-18.5 FIG S5  KOS and n12 virions contain the same viral protein components. MS analysis of viral proteins associated with purified KOS and n12 virions was performed. Download FIG S5, TIF file, 2.2 MB. Copyright © 2018 Dembowski and DeLuca. 2018 Dembowski and DeLuca This content is distributed under the terms of the Creative Commons Attribution 4.0 International license.

**FIG 5:**
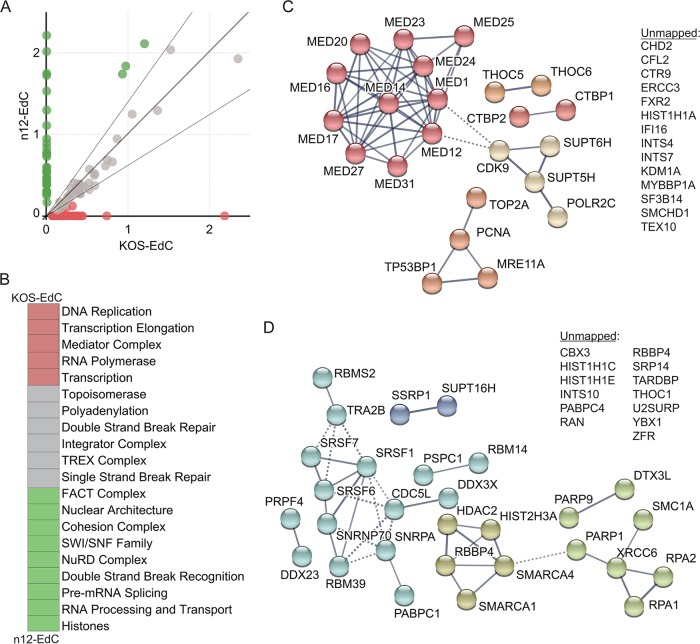
ICP4 is required for the recruitment of host transcription and replication factors to input viral DNA. (A) Relative enrichment of human proteins associated with n12-EdC versus KOS-EdC genomes. Each circle represents an individual viral-genome-associated host protein with the average spectral abundance on KOS-EdC at 3 hpi plotted on the *x* axis and the average spectral abundance on n12-EdC at 3 hpi plotted on the *y* axis. The linear regression line (long, dark gray line) was forced through zero, and the 90% confidence interval is shown by the two shorter, light gray lines. Proteins that fell outside this confidence interval were considered to be relatively more enriched on KOS (red) or n12 (green) viral genomes. (B) Viral-genome-associated host proteins were grouped based on their biological function, and the average SAF values of proteins within each group were compared between KOS-EdC- and n12-EdC-infected cells. Processes that were >1.5-fold more enriched on KOS viral genomes are shown in red, and processes that were >1.5-fold more enriched on n12 viral genomes are shown in green. (C and D) Graphic illustration of predicted physical and functional interactions between human proteins that were relatively more enriched on KOS-EdC viral genomes compared to n12-EdC (C) or n12-EdC viral genomes compared to KOS-EdC (D). See also Table S2 and Fig. S3.

To establish the requirement of ICP4 for the recruitment of host factors to viral DNA, we compared the average SAFs of human proteins enriched on n12 and KOS viral genomes at 3 hpi (Fig. 5A). Proteins that fell within the 90% confidence interval of the linear regression line were considered to be enriched on both KOS and n12 viral genomes. Proteins that fell outside this confidence interval were considered to be enriched on KOS and not n12 viral genomes (red) or enriched on n12 and not KOS viral genomes (green) (Fig. 5A). The identified proteins were further grouped based on their biological function, and the average SAF values of proteins within each group were compared between KOS- and n12-infected cells (Fig. 5B). Processes that were enriched on KOS over n12 genomes are shown in red, and processes that were enriched on n12 over KOS genomes are shown in green (Fig. 5B). To further illustrate the differences in individual proteins that were more enriched on KOS or n12 viral genomes (Fig. 5A), STRING maps were generated (Fig. 5C and D). From these data, we conclude that ICP4 is required for the robust recruitment of the Mediator complex (Fig. 5C, dark red) to viral DNA, as well as several transcription elongation factors (tan; CDK9, SUPT5H, and SUPT6H) and factors that are enriched at viral replication forks (dark orange; TOP2A, PCNA, and MRE11) (15). However, in the absence of ICP4, there is an increase in factors that recognize DNA damage (Fig. 5D, light green; PARP9, DTX3L, PARP1, XRCC6, RPA1, and RPA2), RNA processing factors (light blue), the FACT complex (dark blue; SSRP1 and SPT16H), as well as histones and chromatin remodeling factors (dark green) (Fig. 5D). Consistent with MS results, we did not observe the recruitment of Med23 to n12 viral genomes by immunofluorescence (Fig. 3B). Taken together, ICP4 association with viral DNA triggers a significant change in viral genome architecture resulting in a transition from a state involving recognition as DNA damage to a state associated with robust transcription and viral DNA replication.

### Host proteins associated with input viral genomes at the onset of viral DNA replication.

**FIG 6:**
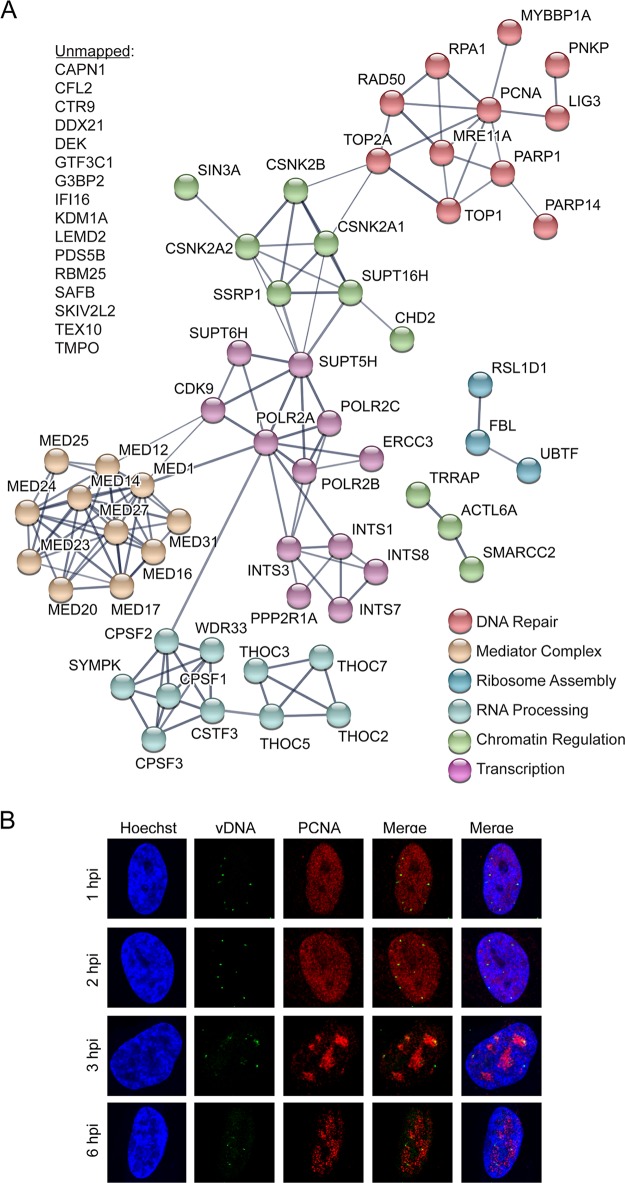
Additional host factors are recruited to input viral DNA at 3 hpi. (A) Graphic illustration of predicted physical and functional interactions between human proteins that associate with KOS-EdC genomes at 3 hpi (MOI of 10 PFU/cell). (B) Input viral genome foci associate with PCNA at 3 hpi. MRC-5 cells were infected with KOS-EdC at an MOI of 10 PFU/cell, fixed at 1, 2, 3, or 6 hpi, and subjected to click chemistry to label input viral DNA (green), Hoechst staining to label nuclei (blue), and indirect immunofluorescence to label PCNA (red). See also Table S2 and Fig. S3.

We previously detected viral DNA replication in infected MRC-5 cells as early as 3 hpi (15). Furthermore, this was the earliest time at which viral replication factors UL30, UL9, and UL42 were detected to associate with input viral DNA (Fig. 1C; Table S2). To investigate host factors that associate with infecting viral genomes immediately after or during the onset of viral DNA replication, host proteins associated with input viral DNA at 3 hpi were identified. At this time, we observed the association of the transcription factor IIH (TFIIH) component ERCC3, the Pol II kinase CDK9, and Mediator component Med31 with viral DNA (Fig. 6A). ERCC3 and CDK9 were previously shown to associate with replicated HSV-1 DNA (15), are known to have roles in the active transcription of cellular genes, and may therefore play a role in activating late viral gene expression. ERCC3, CDK9, and Med31 were not found to associate with viral DNA, at least within the limits of detection by this method, in the absence of ICP4 (Fig. 5C), suggesting that ICP4 may play a role in recruiting these factors.

After 3 h, we also observed the association of PCNA and the topoisomerase subunit TOP2A with viral DNA (Fig. 6A and B). The MRN (MRE11-RAD50-NBS1) double-strand break repair complex members MRE11A and RAD50 also associated at this time (Fig. 6A). Consistent with previous observations, all of these factors have been shown to associate with replicated viral DNA (14, 15). The functions of host repair proteins and PCNA on replicating HSV-1 DNA are not known; however, it has previously been demonstrated that PCNA and MRE11 are required for efficient viral DNA replication (29, 30). Taken together, at the onset of viral DNA replication, another unique set of factors associate with input viral genomes. These factors likely play a role in replication-coupled processes such as the repair of damaged DNA, recombination, or activation of late gene transcription.

### Heterogeneity of genomes at late times postinfection.

**FIG 7:**
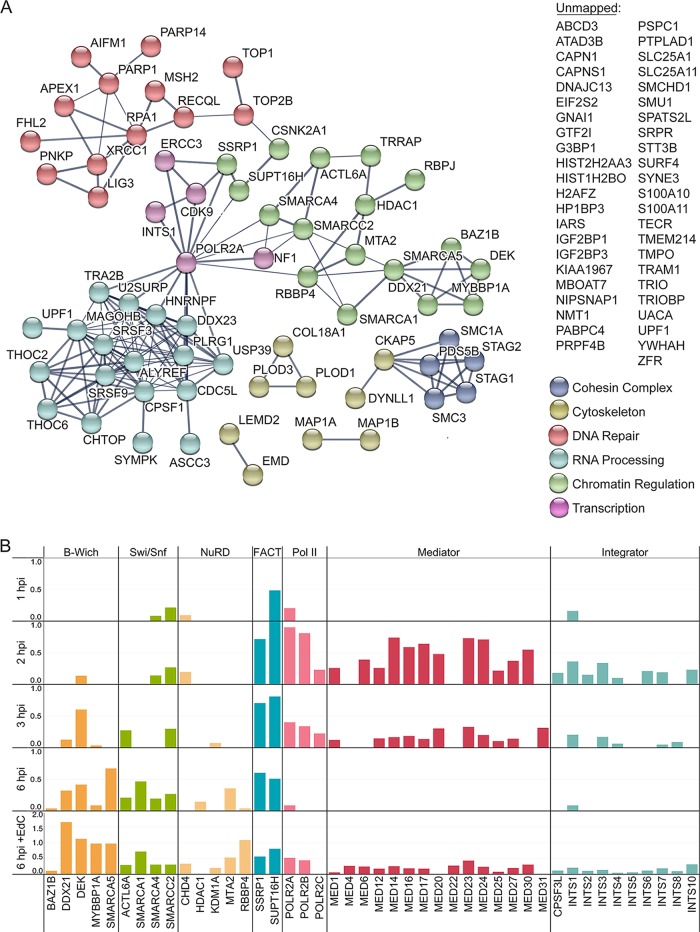
Levels of host transcription factors are selectively reduced on input viral DNA at 6 hpi. (A) Graphic illustration of predicted physical and functional interactions between human proteins that associate with HSV-1 genomes at 6 hpi with KOS-EdC (MOI of 10 PFU/cell). (B) Spectral abundance (SAF) of select viral-genome-associated host proteins that function in transcription regulation. Viral-genome-associated host proteins were detected by MS at 1, 2, 3, or 6 hpi with KOS-EdC. Proteins that associate with viral replication compartments that were labeled with EdC from 4 to 6 hpi (6 hpi + EdC) are also shown (15). Note that KDMA1 is associated with the NuRD complex but is not a core component. See also Table S2 and Fig. S3.

At 6 hpi, we observed the robust association of infecting viral genomes with many host factors (Fig. 7A; Table S2). These factors include the cohesin complex, cytoskeletal proteins, components of the nuclear lamina, DNA repair proteins, RNA processing factors, and factors that regulate chromatin structure. Newly associated proteins include recombination (RECQL), base excision repair (BER) (APEX and XRCC1), and mismatch repair (MMR) (MSH2) proteins, suggesting that viral genomes undergo repair and recombination at this time. Input viral genomes continue to associate with viral replication factors at 6 hpi, and therefore, at least some population of input viral DNA continues to undergo DNA replication. It is likely that BER and MMR occur on nascent viral DNA associated with input viral genomes in the act of DNA replication. Consistent with this hypothesis, we previously observed the association of these factors with replicated viral DNA (14). Another population of viral genomes appear to be packaged into capsids composed of nascent viral proteins (Fig. 1C), and it may also be this population that associates with microtubule-associated proteins (Fig. 7A, MAP1A, MAP1B, DYNLL1, and CKAP5) at this time. Microtubule-associated proteins may facilitate the transport of nascent nucleocapsids. One striking observation is that input genomes exhibit reduced association with several transcription factors, including the Mediator complex, Integrator complex, and Pol II, but increased or continued association with factors that regulate chromatin architecture, including NuRD (nucleosome remodeling and deacetylase), B-WICH, Swi/Snf, and FACT (facilitates chromatin transcription) (Fig. 7B). While the functions of chromatin remodeling factors on viral DNA at this time are unknown, the decrease in Pol II levels suggests that transcription is likely reduced from input viral genomes. Taken together, input viral genomes are present in mixed populations by 6 hpi, whereby some genomes continue to replicate, while others are processed and packaged into virions.

## DISCUSSION

Infecting viral genomes are acted on by host and viral proteins to facilitate sequential steps in infection. Here, we developed and utilized an approach to investigate the origin and temporal association of viral and host proteins with input HSV-1 genomes from nuclear entry through repackaging into nascent capsids within 6 h (Fig. 1B). These studies provide a new and temporally compressed view of the life cycle of HSV-1 based on the dynamics of the genomic proteome and provide new evidence for the involvement of specific host factors in each step.

### Tracking viral protein dynamics.

Early during infection, viral genomes associate with capsid and tegument proteins that originate from the infecting virus (Fig. 1C, 1 to 3 hpi, capsid proteins highlighted in red). However, compared to purified virions (Virion), viral genomes purified after infection are not associated with an abundance of viral glycoproteins and therefore enveloped virus particles. It has previously been demonstrated that the click reaction can access viral genomes only after release from the capsid through the nuclear pore (17) or partial denaturation to disrupt capsid integrity (16). Therefore, at early times, viral genome-associated capsid proteins isolated from nuclei likely associate with viral DNA docked and uncoating at the nuclear pore (see Fig. S2 [1 to 3 hpi] in the supplemental material). Later during infection, nascent capsid proteins that were synthesized after infection and therefore contain heavy amino acids associate with viral genomes (Fig. 1C, 6 hpi) in a similar relative abundance as they constitute intact capsids (Fig. S1B). Therefore, while some of the input genome foci present at 6 h may represent genomes that have not progressed through the infection process as previously proposed (17), our data support a model whereby some population of input viral DNA begins to be repackaged by 6 hpi, putting an upper limit on the minimum time necessary for completion of the nuclear events in productive infection.

Identified interactions with regulatory viral factors are consistent with previous information regarding the virus life cycle. VP16 from the infecting virion associates with viral DNA early during infection (Fig. 1C) to mediate expression of IE viral genes (8, 9). Nascent IE gene products associate with viral genomes as early as 2 hpi (Fig. 1C, IE proteins shown on light gray background). IE gene products, including ICP4, drive the expression of early genes (10–12). Early genes encode components of the viral replication machinery (shown on tan background), which are expressed and associate with viral genomes between 2 and 3 hpi. Consistent with previous observations, ICP8 was the first replication protein to associate (31). By 6 hpi, replication factors are present on input viral genomes in the same relative abundance as in viral replication compartments (Fig. 1C, compare 6 hpi to 6 hpi + EdC), suggesting that another population of the input viral DNA is likely engaged in DNA replication during this time.

### Viral genomes are recognized as DNA damage by the host.

Viral genomes enter into the nucleus as linear, naked DNA containing nicks and gaps (32). The host responds to the invading DNA both by attempting to deposit some form of chromatin on the viral genome and by triggering the recruitment of factors to initiate the repair of damaged DNA. Early during infection, the host also triggers an antiviral response, which is significantly abrogated during infection by the actions of ICP0. We visualized genomes entering into nuclei by 1 hpi (Fig. 1A) and found that these genomes associate with factors whose roles in the intrinsic response to infection have previously been established. These factors includes components of PML NBs (PML, SP100, and SUMO2), which are enriched on viral genomes by 1 hpi (Fig. 2A) but no longer detected by 2 hpi (Fig. 3A). By 2 hpi, PML NBs are dispersed through the actions of ICP0 (5, 33). The effects PML NBs exert on viral DNA are not known. However, PML has been shown to contribute to antiviral repression in the absence of ICP0 (5, 16).

Early during infection, viral genomes also associate with factors that have known roles in the recognition or processing of DNA breaks, including RPA1, PARP1, PARP14, and ligase 3 (LIG3) (Fig. 2A). RPA1 associates with input viral genomes early during infection and remains associated throughout infection (Fig. 2A, 3A, 6A, and 7A). RPA1 has been shown to be recruited to HSV-1 and human cytomegalovirus genomes during infection (34, 35) and is sometimes associated with a subset of PML NBs (36). An interesting observation from these studies is that at least 4× more RPA1 associates with input viral genomes throughout the course of infection compared to replicated viral DNA and 9× more associates with n12 input genomes compared to replicated wild-type viral DNA (Table S2). It is possible that RPA1 binds to genomes that do not progress through the infectious cycle but are present in a repressed state or that there is a unique feature of input genomes, such as nicks and gaps or ends, that enable enhanced binding of RPA1.

PARP1 and PARP14 add poly(ADP-ribose) (PAR) or mono(ADP-ribose) (MAR) groups, respectively, to target proteins. Poly(ADP-ribose) polymerase (PARP) proteins have been implicated in a wide variety of cellular processes, including modification of chromatin, transcription regulation, DNA damage recognition and repair, and promoting inflammatory responses (37). In addition to PARP1 and PARP14, PARP9 and its binding partner DTX3L (an E3 ubiquitin ligase) were found to associate with viral genomes in the absence of ICP4 (Fig. 5D). PARP9 is a catalytically inactive protein that modulates interferon gamma-STAT1 signaling (37). Further analysis of the functions of PARP proteins in viral infection is an important area for future research.

At 2 hpi, we detected IFI16 associated with input viral genomes (Fig. 3) and demonstrate that detected recruitment is dependent on the association of ICP4 with viral DNA (Fig. 5). Previous studies demonstrated that the association of IFI16 with viral DNA coincides with ICP4 recruitment (16, 38). IFI16 binds to HSV-1 DNA and promotes beta interferon signaling (39–41). IFI16 is either directly or indirectly targeted by ICP0 during infection (40, 42). However, we and others were still able to detect IFI16 associated with viral DNA in the presence of ICP0 (39, 43), suggesting that these effects are not absolute. In contrast, we did not observe recruitment to viral DNA of other factors that are known targets of ICP0 including DNA-PKcs, RNF8, and RNF168 (44, 45).

After the onset of viral DNA replication (3 hpi), we detected the topoisomerase TOP2A, MRN complex members MRE11 and RAD50, MMR protein MSH2, BER proteins APEX1 and XRCC1, recombination protein RECQL, and PCNA associated with viral DNA (Fig. 6A and 7A). These data are consistent with our previous observations that TOP2A and MRN complex members are recruited to replicating viral DNA and that MMR proteins and PCNA are recruited to viral replication forks in a replication-dependent manner (14, 15). MRE11, MSH2, and PCNA have previously been shown to be required for HSV-1 DNA replication (29, 30, 46). Furthermore, MRN complex members interact with the viral alkaline nuclease, UL12, and have been proposed to play a role in viral recombination during DNA replication (47).

Together, these data illustrate the timing of association of specific DNA damage response and repair proteins with viral genomes. The time of association likely corresponds to the structure of the viral genome during each stage of infection. Early in infection, the genome contains ends, nicks, and gaps, which are recognized as DNA breaks by host repair proteins. At the same time, factors that mediate intrinsic responses to infection bind. These processes are countered by the actions of ICP0, which disrupts PML NBs and blocks homologous recombination by targeting RNF8 and RNF168 for proteosomal degradation. During DNA replication, the genome is subject to recombination and replication-coupled repair, at which time factors that act in these processes associate. The requirement of some of these factors for productive viral infection has been demonstrated in the past. However, the functional consequences of these interactions are not known.

### A robust transcriptional switch.

IE proteins are expressed and subsequently bind to the viral genome by 2 hpi (Fig. 1C). At this time, we observed the recruitment of several host transcription factors to viral DNA, including the Mediator complex, the Integrator complex, and factors that facilitate cotranscriptional processing of RNA (Fig. 3A). Mediator acts as a transcriptional coactivator of most cellular genes (48). The Integrator complex has multiple roles in host transcription regulation, including promoter proximal pause and release following initiation (49, 50), enhancer RNA biogenesis (51), and snRNA 3′ end formation (52). Integrator has been shown to regulate the processing of herpesvirus saimiri microRNAs (53) and to associate with nascent HSV-1 viral DNA (14, 15). The observation that Integrator is enriched on HSV-1 genomes throughout infection suggests that it plays an important role in viral transcription or cotranscriptional RNA processing.

We demonstrate that robust recruitment of the Mediator complex, Pol II, transcription elongation factors, and replication proteins is ICP4 dependent (Fig. 4 and 5). ICP4 is required for the expression of early viral genes, which encode the viral replication machinery, explaining the role of ICP4 in replication protein recruitment. ICP4 has been shown to interact with TFIID (54) and Mediator (25) and copurifies with factors involved in chromatin remodeling, transcription elongation, and RNA processing (55). We have also shown that ICP4 is required for the binding of components of Mediator and TFIID to viral promoters (25, 56, 57). Here we demonstrate that all Mediator components are either missing or significantly reduced on viral DNA in the absence of ICP4. Therefore, it is likely that the robust recruitment of Mediator by ICP4 drives Pol II recruitment and expression of early and potentially late viral genes. Taken together, ICP4 mediates a robust transcriptional switch that occurs between 1 and 2 hpi to mediate expression of early and potentially late classes of viral genes.

After the onset of DNA replication (3 hpi), another set of transcription factors are recruited, which include the Pol II kinase CDK9, TFIIH component ERCC3, and PAF (polymerase-associated factor) complex member CTR9 (Fig. 6). Recruitment of all of these factors was also dependent on ICP4 (Fig. 5). It is possible that these factors may play some role in promoting late gene expression after the onset of viral DNA replication or that this switch may be mediated by some change in viral genome architecture that occurs at this stage of infection. Alternatively, these and other factors that were not detected earlier during infection may have fallen below the limits of detection by this assay.

Interestingly, by 6 hpi, there is a dramatic decrease in viral-genome-associated transcription factors, including Pol II, Mediator, and Integrator (Fig. 7B). Perhaps at this time transcription is reduced on input genomes to facilitate viral DNA packaging and/or DNA replication. However, there is still an abundance of RNA processing factors present. This is consistent with replication fork pulse-chase data (15), in which transcription factors were more enriched on replication forks and RNA processing factors were more abundant on nascent viral DNA. These data may provide insight into the mechanism of replication coupled to late gene transcription, suggesting that the initiation of transcription is closely linked to an act of DNA replication.

Another abundant group of proteins associated with viral genomes throughout infection are factors that regulate chromatin structure, including the B-Wich, Swi/Snf, NuRD, and FACT complexes (Fig. 7B). In general, the abundance of factors that regulate chromatin increase on input genomes with time. Perhaps these factors function to remove histones from replicated viral genomes or keep them from binding in the first place, allowing for robust late gene expression.

Although core histones consistent with the presence of nucleosomes were not detected to associate with purified input genomes, individual histone proteins were detected to some extent. It is possible that this approach is limited in the ability to detect histones or that histones are less stably bound to viral DNA during productive infection compared to host DNA (58).

### A powerful approach to investigate viral infection.

In this study, we present a new and powerful approach to investigate viral infection. We define the stages of the HSV-1 life cycle by the sets of viral and cellular proteins that associate with the viral genome. These stages were defined from the perspective of the infecting viral genome, which because DNA replication is semiconservative, could be tracked from when it first uncoats until it is packaged in progeny virions. We also utilized a virus that does not express ICP4 (n12) to identify host factors associated with a robust transcriptional switch that mediates early viral gene expression. Information regarding viral genome dynamics not only provide new insight into the involvement of viral and host proteins in processes that occur on viral DNA but also can lead to the development of new antiviral agents that target these proteins.

## MATERIALS AND METHODS

### Cells and viruses.

Experiments were performed using MRC-5 human embryonic lung (CCL-171) or Vero African green monkey kidney (CCL-81) cells obtained from and propagated as recommended by ATCC. The viruses used in this study include the wild-type HSV-1 strain, KOS, and the ICP4 mutant virus, n12 (28). n12 virus stocks were prepared, and the titers of the virus in the Vero cell-based ICP4-complementing cell line E5 were determined.

### Virus purification for analysis of virion-associated proteins.

Confluent monolayers of Vero or E5 cells (2 × 10^8^ cells) were infected with KOS or n12 virus, respectively, at a multiplicity of infection (MOI) of 5 PFU/cell. After 24 h, infected cells were scraped into the medium. The medium containing the infected cells was adjusted to 0.5 M NaCl and incubated on ice for 45 min. The cells were pelleted at 3,000 × *g* for 15 min at 4°C. The supernatant was then filtered through a 0.8-µm filter, and the filtrate was centrifuged at 25,000 × *g* for 2 h at 4°C. The virus-containing pellets were allowed to resuspend overnight in Tris-buffered saline (TBS). Benzonase was added to the virus sample, which was allowed to incubate for 30 min at 37°C. The virus was then layered onto a preformed 30 to 65% (wt/vol) sucrose gradient. The gradients were centrifuged in an SW41Ti rotor at 20,000 rpm overnight at 4°C. One-milliliter fractions were collected from the bottom of the tube. Ten microliters of each fraction were incubated overnight in 90 µl of 0.6% SDS and 400 µg/ml proteinase K at 37°C. The digested samples were diluted and assayed for viral DNA by real-time (RT)-PCR. The peak of viral DNA corresponded to fractions just below the middle of the tube, which also corresponds to the density of HSV-1. The peak fractions were diluted with TBS and centrifuged in the SW41Ti rotor at 24,000 rpm for 2 h at 4°C. The supernatant was discarded, and the pellets were allowed to resuspend in a small volume overnight at 4°C. Virus titer and genome number were determined by plaque assay and RT-PCR, respectively. Viral proteins were denatured in SDS sample buffer, and viral protein constituents were determined by MS (Fig. 1C [Virion], Fig. S5, and Table S2 [Virion]).

### Preparation of EdC-labeled virus stocks.

Confluent monolayers of 2 × 10^8^ Vero or E5 cells were infected with KOS or n12 virus, respectively, at an MOI of 10 PFU/cell at 37°C for 1 h. After the monolayers were rinsed with TBS to remove unadsorbed virus, the medium was replaced with Dulbecco’s modified Eagle medium (DMEM) containing 5% fetal bovine serum (FBS). Four hours later, 5-ethynyl-2′-deoxycytidine (EdC) was added at a final concentration of 5 to 10 µM. Monolayers were harvested 34 to 36 h after infection, freeze-thawed three times at −80°C, sonicated, and clarified by low-speed centrifugation. Viral supernatants were passed over a G-25 column to remove residual EdC. Viral titers were determined by plaque assay on Vero or E5 cells, and viral genome number was determined by RT-PCR using primers specific for the viral thymidine kinase gene as described previously (20) (Table S1).

### Viral genome imaging and immunofluorescence.

A total of 2 × 10^5^ Vero or MRC-5 cells were grown on glass coverslips in 12-well dishes. Infections were carried out using EdC-labeled virus stocks at an MOI of 10 PFU/cell, and click chemistry and immunofluorescence were conducted as described previously (14). ICP4 antibodies include the mouse monoclonal antibody 58S (Fig. 1A and Fig. S4) (59) and the rabbit polyclonal antibody N15 (Fig. S2). The monoclonal antibody 58S recognizes only the dimeric form of ICP4, which binds to viral DNA (60). Images were obtained using an Olympus Fluoview FV1000 confocal microscope. For images in Fig. 1A, background subtraction and subsequent deconvolution of each Z stack was performed manually using Huygens Essential software (Scientific Volume Imaging BV). Imaris software (Bitplane AG) was used for image rendering.

### SILAC labeling and affinity purification of viral genomes.

Prior to infection, MRC-5 cells were propagated for at least three passages in medium containing heavy amino acids (l-arginine ^13^C_6_^15^N_4_ and l-lysine ^13^C6 ^15^N_2_), while virus stocks used to infect the cells were prepared in the presence of nonisotopically labeled or light amino acids. Confluent monolayers (~7 × 10^7^) of these cells were infected with EdC-labeled KOS or n12 virus at an MOI of 10 PFU/cell for 1 h at room temperature. After adsorption, the inoculum was removed, and cells were rinsed with room temperature TBS before SILAC growth medium was replaced. Cells were incubated at 37°C for 1 to 6 h. For each sample there was a corresponding negative control, in which SILAC cells were infected with virus that was not prelabeled with EdC using the same infection conditions. Harvesting nuclei from infected cells, biotin conjugation to EdC-labeled viral genomes by click chemistry, nuclear lysis, DNA fragmentation, streptavidin purification, and elution of associated proteins were conducted as described previously (15, 19).

### Mass spectrometry and data analysis.

MS was carried out by MS Bioworks as described previously (14). For protein identification, data were searched using Mascot and Mascot DAT files were parsed into the Scaffold software for validation and filtering and to create a nonredundant list per sample. Data were filtered at 1% protein and peptide level false-discovery rate (FDR) and requiring at least two unique peptides per protein. Viral proteins with at least five spectral counts (SpCs), enriched by at least twofold over the SpC for the unlabeled negative control, and present in two biological replicates were considered to be enriched on viral DNA (Table S2). In cases where no SpCs were detected in the negative control, the denominator was set at 1 to determine the fold enrichment of viral-genome-associated proteins. Host factors with at least five SpCs, enriched by at least threefold over the SpC for the unlabeled negative control, and present in two biological replicates were considered to be enriched on viral DNA. For human proteins that are common contaminants of affinity purification-MS data sets (61), the threshold for confident detection was increased to a fivefold relative enrichment compared to the unlabeled negative control.

For SILAC analysis, data were processed through MaxQuant software 1.5.1.0 to recalibrate MS data, filter the database at the 1% protein and peptide FDR, and to calculate SILAC heavy/light (H/L) ratios. Proteins were distinguished as either heavy or light based on a twofold enrichment of heavy or light peptides, respectively. Proteins that fell between this range were labeled as intermediate (I). Identified proteins were displayed graphically using GraphPad Prism or Tableau software. Potential physical and functional protein-protein interactions among proteins identified were illustrated using the STRING protein-protein interaction network database (23). String diagrams were modified in Adobe Illustrator for optimal data presentation.
