# Supplementary material for: Temporal Viral Genome-Protein Interactions Define Distinct Stages of Productive Herpesviral Infection
Source: mBio. 2018 Jul 17;9(4):e01182-18. doi: 10.1128/mBio.01182-18 (PMC6050965; doi:10.1128/mBio.01182-18)
Supplement: TABLE S1 [file mbo004183982st1.doc]

| **Virus** | **Genomes/μL** | **PFU/μL** | **Genome/PFU** |
| --- | --- | --- | --- |
| KOS | 5.48 x 10^8^ | 1.90 x 10^7^ | 29/1 |
| KOS-EdC | 2.09 x 10^8^ | 2.38 x 10^6^ | 88/1 |
| n12 | 5.17 x 10^8^ | 1.40 x 10^7^ | 37/1 |
| n12-EdC | 2.24 x 10^8^ | 3.00 x 10^6^ | 75/1 |

**Table S1. The Effects of EdC Labeling on Viral Genome to PFU Ratio.** Virus stocks were prepared in the presence or absence of EdC (KOS-10 μM, n12-5 μM final concentration) and genome number and PFU were determined by real-time PCR and plaque assay, respectively. n12 virus stocks were prepared and titered in the ICP4 complementing cell line, E5. Values indicate the number of genomes or PFU per μL of virus stock.
