# Supplementary figures and images for: Temporal Viral Genome-Protein Interactions Define Distinct Stages of Productive Herpesviral Infection
Source: mBio. 2018 Jul 17;9(4):e01182-18. doi: 10.1128/mBio.01182-18 (PMC6050965; doi:10.1128/mBio.01182-18)

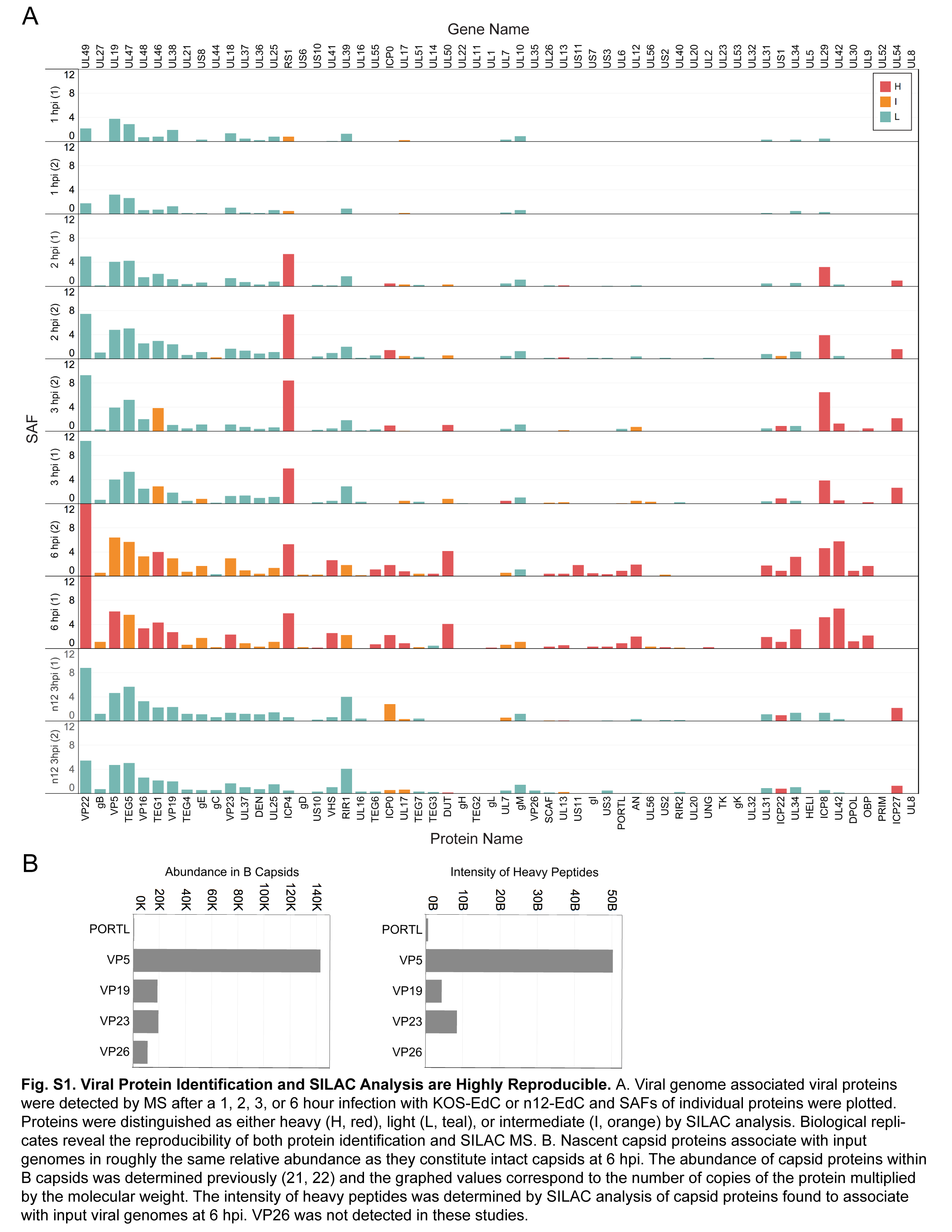

Supplement: FIG S1 [file mbo004183982sf1.tif]

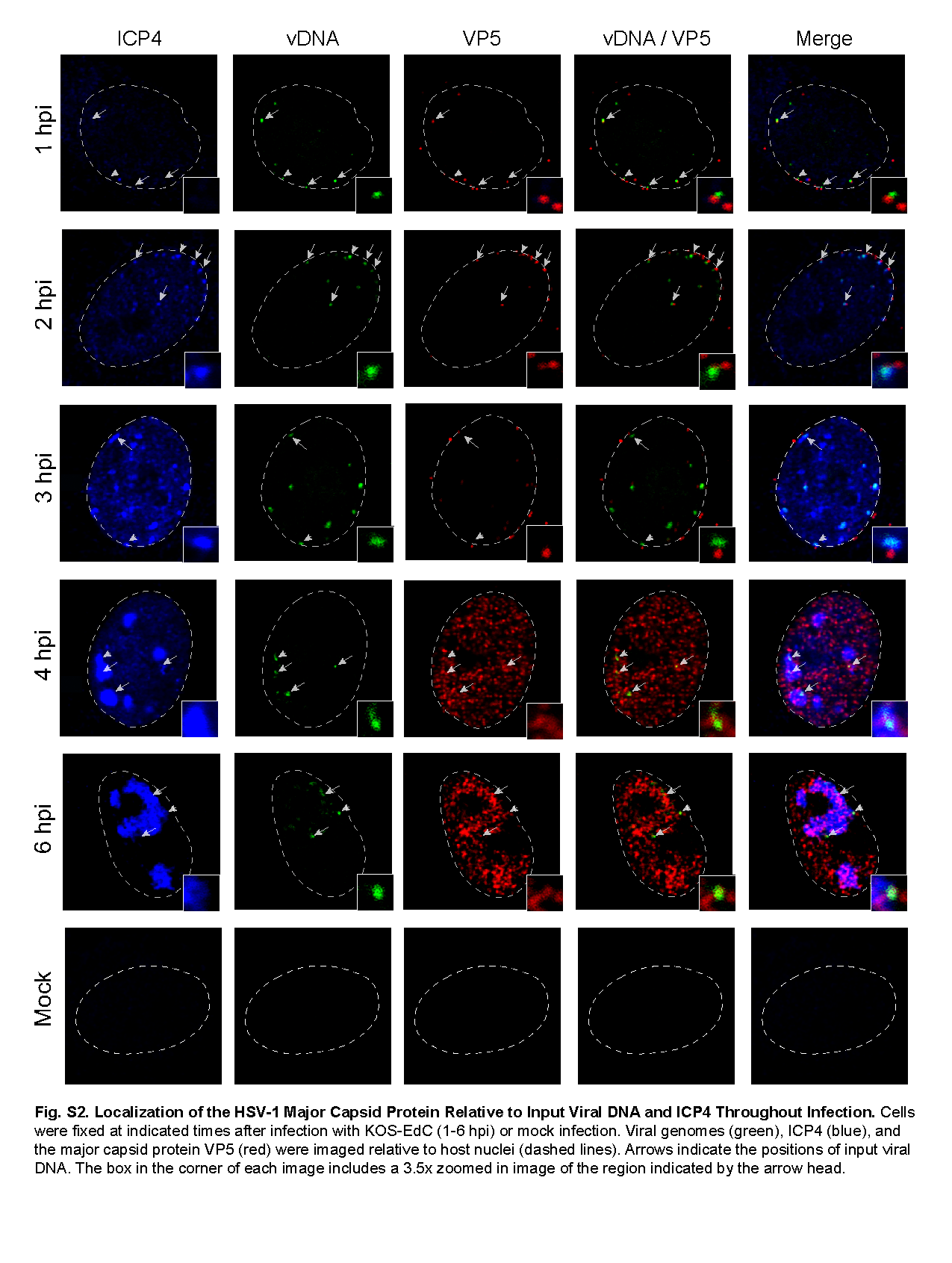

Supplement: FIG S2 [file mbo004183982sf2.tif]

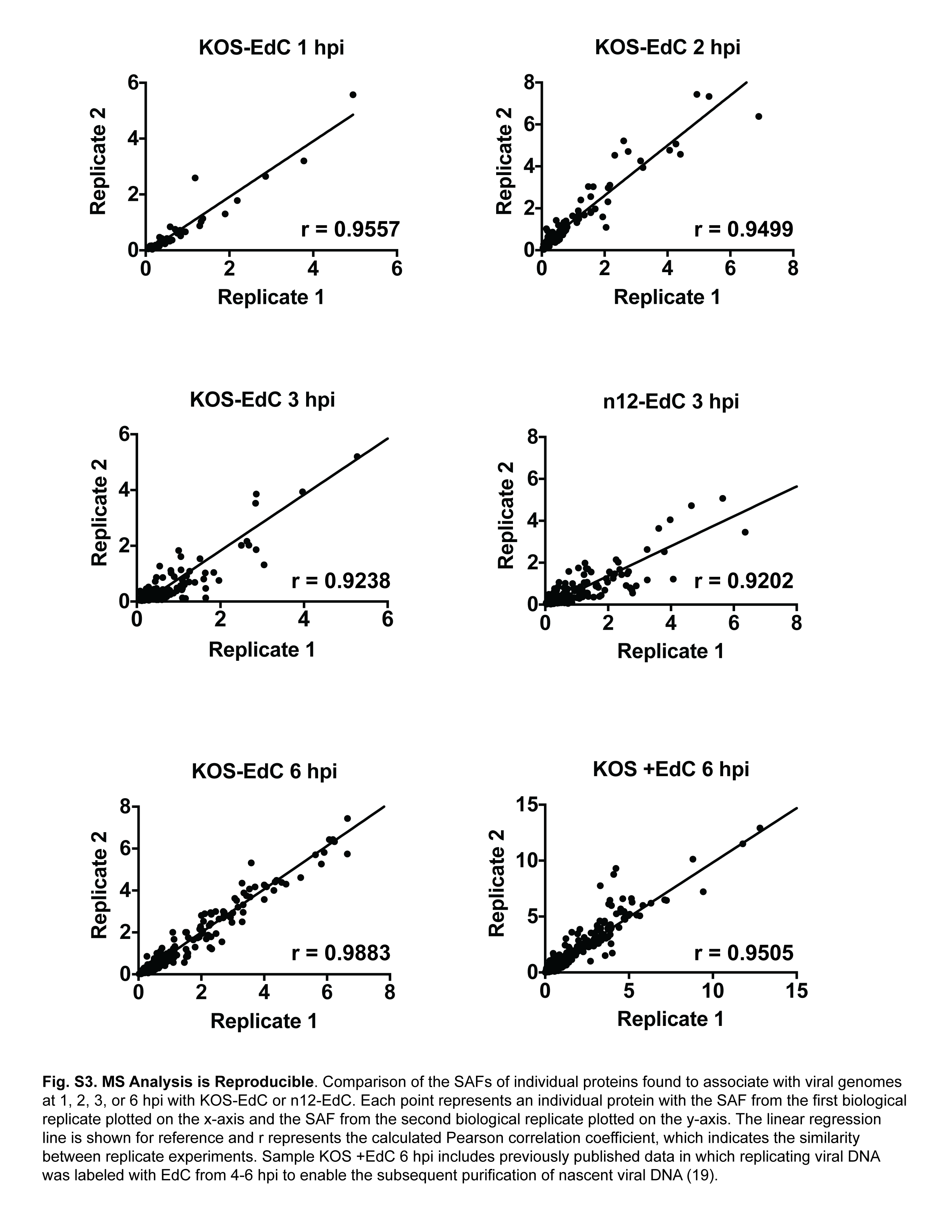

Supplement: FIG S3 [file mbo004183982sf3.tif]

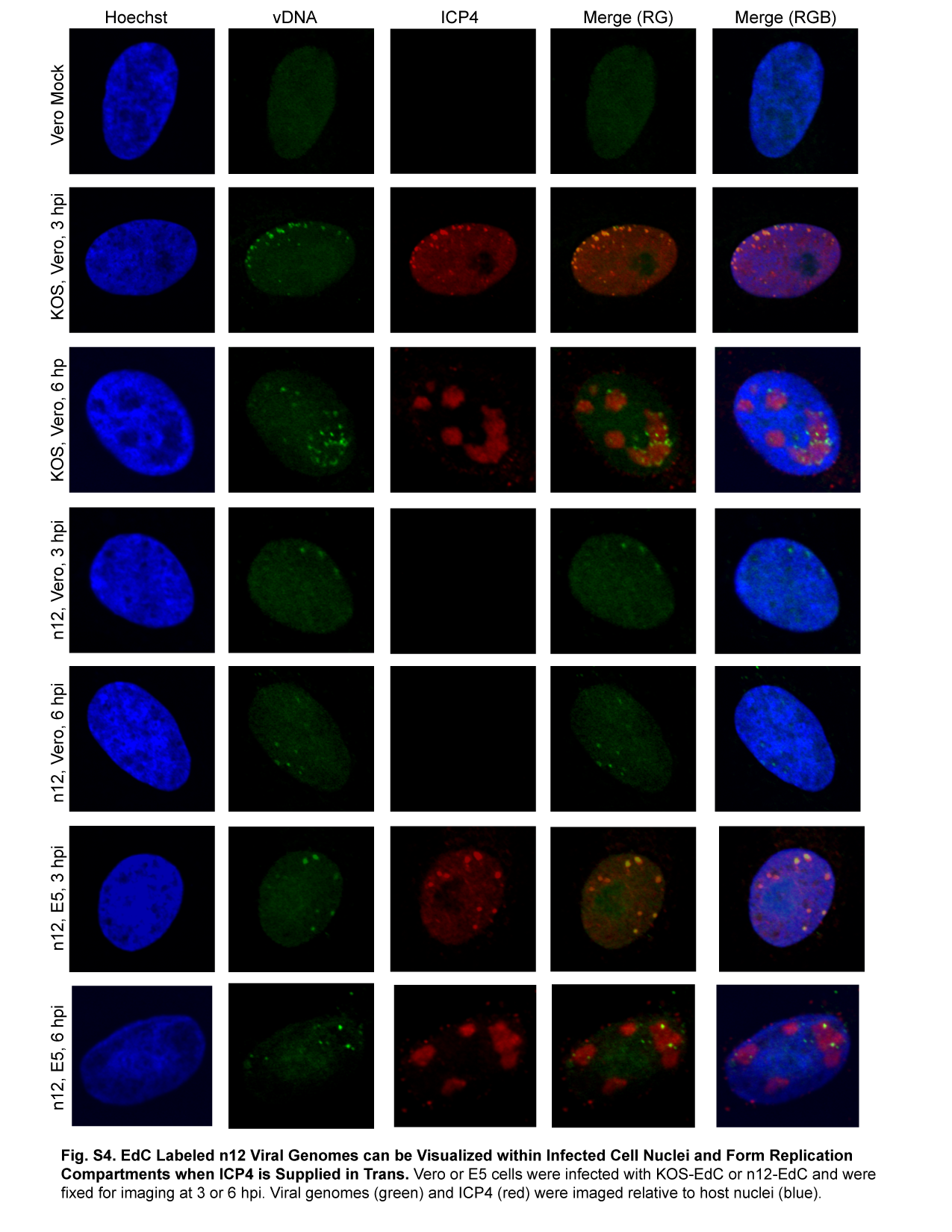

Supplement: FIG S4 [file mbo004183982sf4.tif]

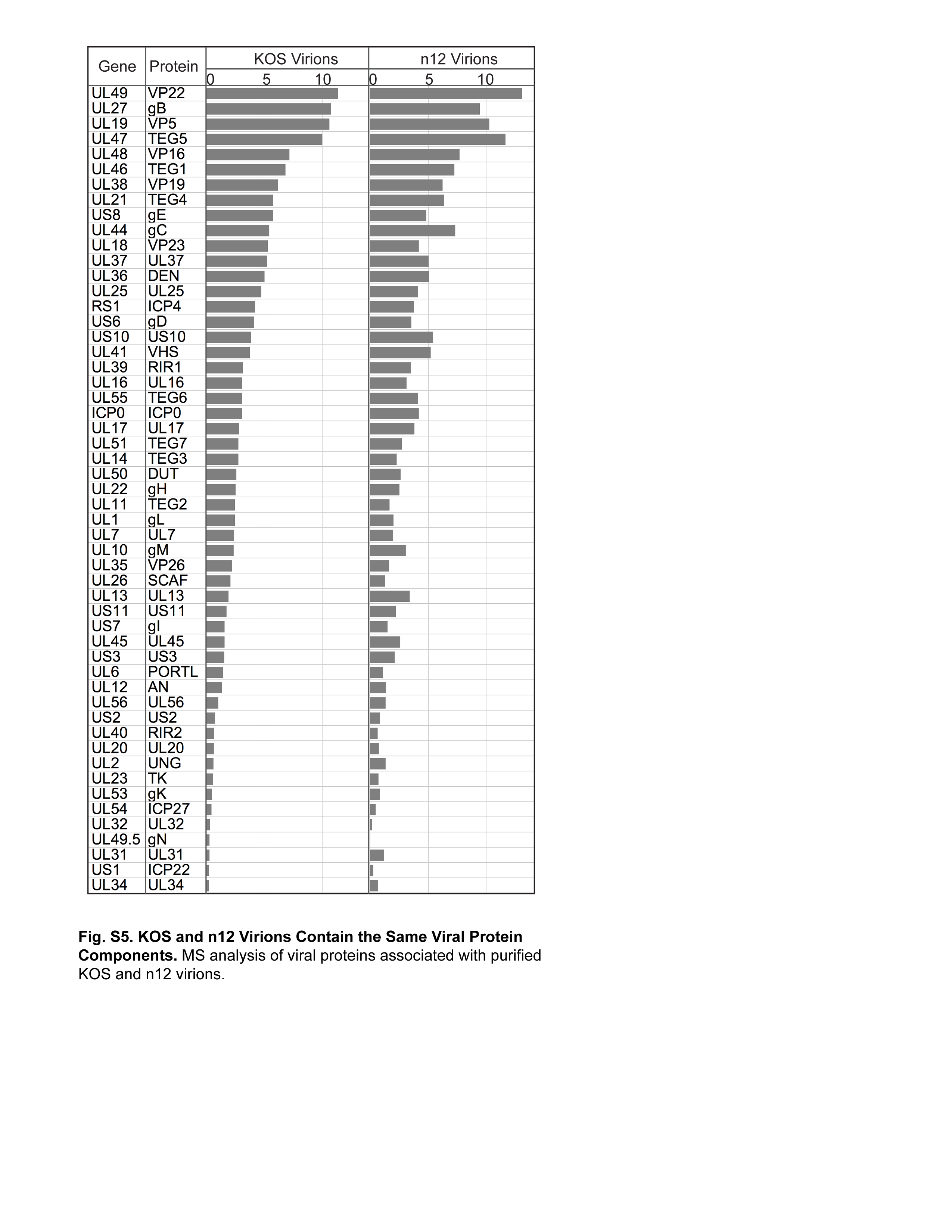

Supplement: FIG S5 [file mbo004183982sf5.tif]
